# Parents’ role as advocates for and against childhood vaccination: Findings from a nationally representative survey

**DOI:** 10.1371/journal.pone.0354147

**Published:** 2026-07-23

**Authors:** Kathryn L. Kennedy, Tara L. Queen, Mallory K. Ellingson, Emma H. Elias, William A. Calo, Nora E. Rosenberg, Noel T. Brewer, Melissa B. Gilkey

**Affiliations:** 1 Department of Health Behavior, Gillings School of Global Public Health, University of North Carolina, Chapel Hill, North CarolinaUnited States of America; 2 Lineberger Comprehensive Cancer Center, University of North Carolina, Chapel Hill, North Carolina, United States of America; 3 School of Medicine, Duke University, Durham, North Carolina, United States of America; 4 Department of Public Health Sciences, Penn State College of Medicine, Hershey, Pennsylvania, United States of America; Federal University Otuoke, NIGERIA

## Abstract

**Introduction:**

In 2025, the US recorded the highest number of measles cases in 25 years, signaling an urgent need for renewed attention to increasing childhood vaccination coverage. To identify future intervention partners, we sought to understand parents’ role in advocating for and against childhood vaccines.

**Methods:**

A nationally representative sample of 1,315 parents of children ages 9–12 years participated in our online survey about vaccine advocacy behaviors in 2025. Separate weighted logistic regression models identified correlates of encouraging or discouraging other parents from getting routine vaccines for their children.

**Results:**

Over one-third of parents reported encouraging routine vaccines sometimes or more often (36%), while about one-tenth reported discouraging vaccines (12%). These estimates include a small proportion of parents (6%) who reported both behaviors. Parents with higher confidence in the importance of vaccines for their child’s health were more likely to be encouragers and less likely to be discouragers (both *p* < .05). Encouragers (*n* = 477) most often advocated for measles (50%) or seasonal flu vaccines (44%). These parents most often engaged family members and close friends, using strategies such as sharing vaccine experiences (47%), information about vaccine effectiveness (38%), and advice or opinions about vaccines (35%). Discouragers (*n* = 151) most often advocated against Covid-19 (53%) or seasonal flu vaccines (41%), with relatively few focusing on measles vaccine (10%). Discouragers engaged similar audiences and strategies as encouragers.

**Conclusion:**

Findings of our nationally representative study suggest that parents who encourage routine vaccination far outnumber those who discourage it. Given that encouragers often advocate for measles vaccine, future efforts to increase vaccination coverage should consider ways to partner with parent advocates to stem the alarming resurgence of this disease.

## Introduction

The United States (US) recorded over 2,200 measles cases in 2025, constituting the highest number of cases since measles was declared eliminated in 2000 [1,2]. This resurgence follows several years of small declines in coverage for measles, mumps, and rubella (MMR) vaccination. Among children enrolled in kindergarten, coverage dropped from the Healthy People 2030 goal of 95% during the 2019–2020 school year to 93% during 2024–2025 [1,3]. This coverage is characterized by pronounced geographic disparities, ranging from 98% in Connecticut to just 80% in Idaho, with fewer than one-quarter of states meeting the 95% threshold needed to maintain community immunity [3,4]. While measles is perhaps the most dramatic example, kindergarten coverage for other childhood vaccines, including diphtheria, tetanus, and acellular pertussis (DTaP), polio, and varicella, have seen similar declines, from 95% to 92–93%, over the past five school years [3,4]. There is also concern for adolescent vaccines as HPV vaccination coverage stagnated for the third year in 2024, with only 63% of 13- to 17-year-olds up to date on the multi-dose series [5].

Taken together, these data suggest that renewed attention to childhood vaccination coverage is urgently needed to reverse these concerning trends. A promising direction is engagement of social networks with the broad reach needed to address the national scope of the problem across multiple vaccine types [6]. Primary care providers have long served as champions for routine childhood vaccination, but investment in other trusted messengers may be valuable in light of post-pandemic declines in trust of physicians and healthcare organizations [7–10].

Engaging parent advocates and their social networks may offer another way to increase coverage for MMR and other routine childhood vaccines. Parents have the potential to advocate for vaccination by sharing resources, social media posts, and personal stories, having conversations with other parents, and leading community campaigns [11–14]. On the one hand, prior research suggests that encouragement from family members, friends, and social networks is a predictor of vaccine acceptance, and over 80% of parents trust these peers for vaccine-related advice [11,15,16]. For example, parents report improved vaccine-related attitudes after engaging in conversations promoting vaccination with parent advocates, and social network analysis suggests such “people networks” predict parents’ own vaccination behavior [11,15]. On the other hand, parents may also discourage vaccination given their own beliefs, attitudes, and experiences [17]. For example, even among parents who receive a provider recommendation, HPV vaccine refusal is higher for those exposed to anti-HPV vaccination advice from social contacts, suggesting that parents’ influence cuts both ways through encouraging or discouraging vaccination [15,16]. To our knowledge, no prior nationally representative studies have compared these advocacy behaviors to understand how common they are or what they entail.

We sought to address this gap by examining parents’ role in advocating for and against routine childhood vaccines. Our study aim was to evaluate the prevalence and correlates of encouraging or discouraging childhood vaccination to other parents. We also sought to compare encouragers and discouragers on the vaccine types, audiences, strategies, and modes they engaged, as well as on the advocacy identities they endorsed. In this way, our study seeks to advance opportunities to engage parent advocates in future efforts to increase vaccination coverage.

## Methods

### Participants

Participants were US parents and other caregivers (hereafter “parents”) of at least one 9- to 12-year-old child who were participating in a broader cross-sectional online survey about adolescent health. Participants were members of an existing panel of community-dwelling adults constructed from a probability-based sample of households using phone- and address-based sampling frames designed to be representative of the US population [18]. Of the 2,488 parents in the initial random sample from the panel, 1,569 responded and completed screening questions to confirm their eligibility (response rate 63%). We excluded 254 parents who took the survey in less than one-third of the median completion time of 16 minutes or did not answer more than half of the survey items. Data were not available to compare included and excluded parents. Our final sample consisted of 1,315 parents.

### Procedures

We conducted our survey in May 2025. The initial invitation to participate in the survey was via e-mail, text message, or telephone, followed by up to two reminders for survey completion. Participants provided web-based informed consent prior to starting the survey and received points that they could apply towards incentive rewards. Parents with more than one eligible child answered survey items about the child with the most recent birthday. The University of North Carolina Institutional Review Board approved the study protocol.

### Measures

#### Parent advocacy behaviors.

Participants completed two randomly ordered survey blocks of measures related to encouraging and discouraging vaccines. The survey block on encouraging vaccines began with an introduction to the topic: “Some people feel strongly that parents should get one or more routine vaccines for their children. These people may encourage others to vaccinate by sharing information, giving advice, posting on social media, or participating in community events.” To assess advocacy frequency*,* the survey item then read: “How often have you encouraged other parents to get routine vaccines for their children?” accompanied by a 5-point response scale. We categorized parents as *vaccine encouragers* if they responded “sometimes”, “often”, or “all the time” versus not vaccine encouragers if they responded “never” or “rarely”.

The survey block on discouraging vaccines began with: “Some people feel strongly that other parents should not get one or more routine vaccines for their children. These people may discourage others from vaccinating by sharing information, giving advice, posting on social media, or participating in community events.” The survey item then read: “How often have you discouraged other parents from getting routine vaccines for their children?” with the same 5-point response scale described above. We used the same coding strategy as for vaccine encouraging to categorize parents as *vaccine discouragers*.

#### Vaccine advocacy role.

Within each survey block, participants categorized as encouragers or discouragers completed four items about their role advocating for or against routine childhood vaccination. One item assessed the *types of vaccines* parents advocated about using a pre-specified list (measles or MMR, meningitis, seasonal flu, HPV, Covid-19, other routine vaccines for children, all of the above). The three remaining items used prespecified lists to assess parents’ advocacy *audiences* (close friends, family members, other parents at my child’s school or in my community, other parents on social media, the broader public), advocacy *strategies* (sharing experiences getting vaccines for children, information about vaccine effectiveness, advice or opinions about vaccines, information about vaccine safety, websites or other resources to learn about vaccines, information about which providers to go to), and advocacy *modes* (conversations with other parents, social media posts, school or community advocacy, state or national advocacy).

#### Advocacy identities.

All participants answered an item about advocacy *identities*, selecting any of seven terms they would use to describe themselves (vaccine advocate, child health advocate, wellness advocate, vaccine skeptic, medical freedom activist, anti-vaccine activist, or vaccine influencer).

#### Parent characteristics.

Three items assessed parent characteristics including their parenting role, political leaning, and perceived importance of vaccines for their child’s health (hereafter “vaccination confidence”). The survey company provided data on the parent’s race/ethnicity, educational attainment, household income, and US census region.

#### Survey development.

We used a combination of new survey items developed by our team and items adapted from previous research about parents, vaccine advocates, and health communication [8,11,13,19–22]. We conducted cognitive interviews for the new items with a convenience sample of seven parents to improve item wording and clarity before beginning data collection. We also pre-tested the survey instrument with 25 parents from the national panel to check the functionality of survey features such as skip patterns.

### Data analysis

We report unweighted frequencies and weighted percentages, applying post-stratification survey weights to generate nationally representative estimates. To identify correlates of vaccine encouraging and discouraging, we conducted separate bivariate and multivariable logistic regressions for each outcome. We selected candidate correlates based on their availability and association with vaccine uptake and related cognitions and behaviors, resulting in the inclusion of parenting role, [5,23,24] race/ethnicity, [25–27] educational attainment, [24] political leaning, [28] vaccination confidence, [23,29,30] and household income [23,25,31].

We compared vaccine encouragers to discouragers on their advocacy roles (vaccine types, audiences, strategies, and communication mode) using generalized estimating equations with repeated measures. We treated each binary outcome as a repeated measure as parents had the opportunity to both encourage and discourage. We conducted analyses using Stata Version 19.0 (College Station, TX) and SAS Version 9.4 (Cary, NC). Statistical tests were two-tailed with a critical alpha of 0.05.

## Results

### Sample characteristics

Participants were mothers (56%), fathers (43%), or other guardians (1%, Table 1). Most identified as non-Hispanic White (55%), Hispanic (23%), or non-Hispanic Black (12%). More than one-third of parents had a bachelor’s degree or more education (42%). Political leaning was split across conservative (34%), moderate (43%), and liberal (23%) views. Household income ranged from low (<$50,000, 19%) to medium ($50,000-$99,999, 25%) to high (≥$100,000, 56%). Over one-third of participants lived in the South (40%), with the remainder in the West (23%), Midwest (21%), or Northeast (16%). Over one-half of parents reported high vaccination confidence (57%).

**Table 1:** Sample characteristics (*n* = 1,315).

	*n*	(%)
Parenting role		
Mother	804	(56)
Father	499	(43)
Grandparent or other guardian	12	(1)
Race/ethnicity		
Non-Hispanic White	803	(55)
Non-Hispanic Black	198	(12)
Hispanic	201	(23)
Other^a^	113	(11)
Educational attainment		
Some college or less	641	(58)
Bachelor’s degree or higher	674	(42)
Political leaning		
Conservative	446	(34)
Moderate	552	(43)
Liberal	317	(23)
Household income		
Low (<$50,000)	295	(19)
Medium ($50,000-$99,999)	344	(25)
High (≥$100,000)	676	(56)
US Census region		
Northeast	189	(16)
Midwest	318	(21)
South	500	(40)
West	308	(23)
Vaccination confidence		
Low (vaccine are moderately, a little, or not at all important)	560	(43)
High (vaccines are very important)	755	(57)

Table shows unweighted frequencies and weighted percentages. Percentages may not sum to 100% due to rounding.

^a^Includes non-Hispanic 2 + races (*n* = 35) and non-Hispanic other (*n* = 78).

### Prevalence

Parents who reported engaging in vaccine advocacy behaviors sometimes or more often included those who only encouraged vaccines to other parents (29%), only discouraged vaccines (6%), and both encouraged and discouraged vaccines (6%). Vaccine encouragers, who reported encouraging vaccines either alone or in combination with discouraging (*n* = 477), consisted of those who encouraged vaccines sometimes (58%), often (23%), and all the time (19%). Vaccine discouragers, who reported discouraging vaccines either alone or in combination with encouraging (*n* = 151), consisted of those who discouraged vaccines sometimes (74%), often (15%), and all the time (11%).

### Correlates of vaccine advocacy

Fathers and other guardians were less often vaccine encouragers compared to mothers (32% vs. 39%, *p* < .05, Table 2). Parents with moderate and liberal versus conservative political leanings were more often encouragers (38% and 50% vs. 24%, *p* < .05), as were parents with high versus low vaccination confidence (48% vs. 20%, *p* < .05). These multivariable findings mirrored those of bivariate analyses (S1 Table).

**Table 2:** Multivariable correlates of vaccine encouraging and discouraging (*n* = 1,315).

	No. of parents who encourage/ No. of parents in category (%)		Multivariable		No. of parents who discourage/ No. of parents in category (%)		Multivariable	
			OR	(95% CI)			OR	(95% CI)
Parenting role								
Mother	310/804	(39)	1		89/804	(11)	1	
Father, grandparent or other	167/511	(32)	0.71	(0.52-0.97)*	62/511	(14)	1.33	(0.85-2.06)
Race/ethnicity								
Non-Hispanic White	294/803	(35)	1		76/803	(10)	1	
Non-Hispanic Black	70/198	(32)	0.83	(0.52-1.32)	31/198	(20)	2.15	(1.13-4.07)*
Hispanic	72/201	(37)	0.97	(0.64-1.47)	30/201	(16)	1.65	(0.96-2.85)
Other	41/113	(41)	1.11	(0.68-1.82)	14/113	(11)	1.56	(0.76-3.20)
Educational attainment								
Some college or less	218/641	(34)	1		92/641	(15)	1	
Bachelor’s degree or higher	259/674	(39)	1.06	(0.75-1.49)	59/674	(8)	0.66	(0.41-1.06)
Political leaning								
Conservative	109/446	(24)	1		65/446	(14)	1	
Moderate	203/552	(38)	1.65	(1.17-2.34)*	68/552	(15)	1.02	(0.62-1.67)
Liberal	165/317	(50)	1.98	(1.33-2.93)*	18/317	(5)	0.53	(0.26-1.06)
Vaccination confidence								
Low	111/560	(20)	1		111/560	(20)	1	
High	366/755	(48)	3.06	(2.19-4.27)**	40/755	(6)	0.33	(0.20-0.54)**
Household income								
Low	113/295	(37)	1		49/295	(18)	1	
Medium	108/344	(32)	0.75	(0.47-1.21)	42/344	(13)	0.83	(0.44-1.56)
High	256/676	(37)	0.92	(0.58-1.46)	60/676	(10)	0.81	(0.45-1.48)

Table shows unweighted frequencies and weighted percentages and odds ratios. OR: odds ratio. CI: confidence interval.

*p < 0.05.

**p < 0.001.

Non-Hispanic Black parents were more often vaccine discouragers compared to non-Hispanic White parents (20% vs. 10%, *p* < .05, Table 2). Parents with high versus low vaccination confidence were less often discouragers (6% vs. 20%, *p* < .05). In bivariate analyses, vaccine discouraging was also associated with lower educational attainment, conservative versus liberal political leaning, and lower household income, yet these associations did not retain statistical significance in multivariable analyses (S1 Table).

### Vaccine advocacy role

#### Vaccine types.

**Fig 1:**
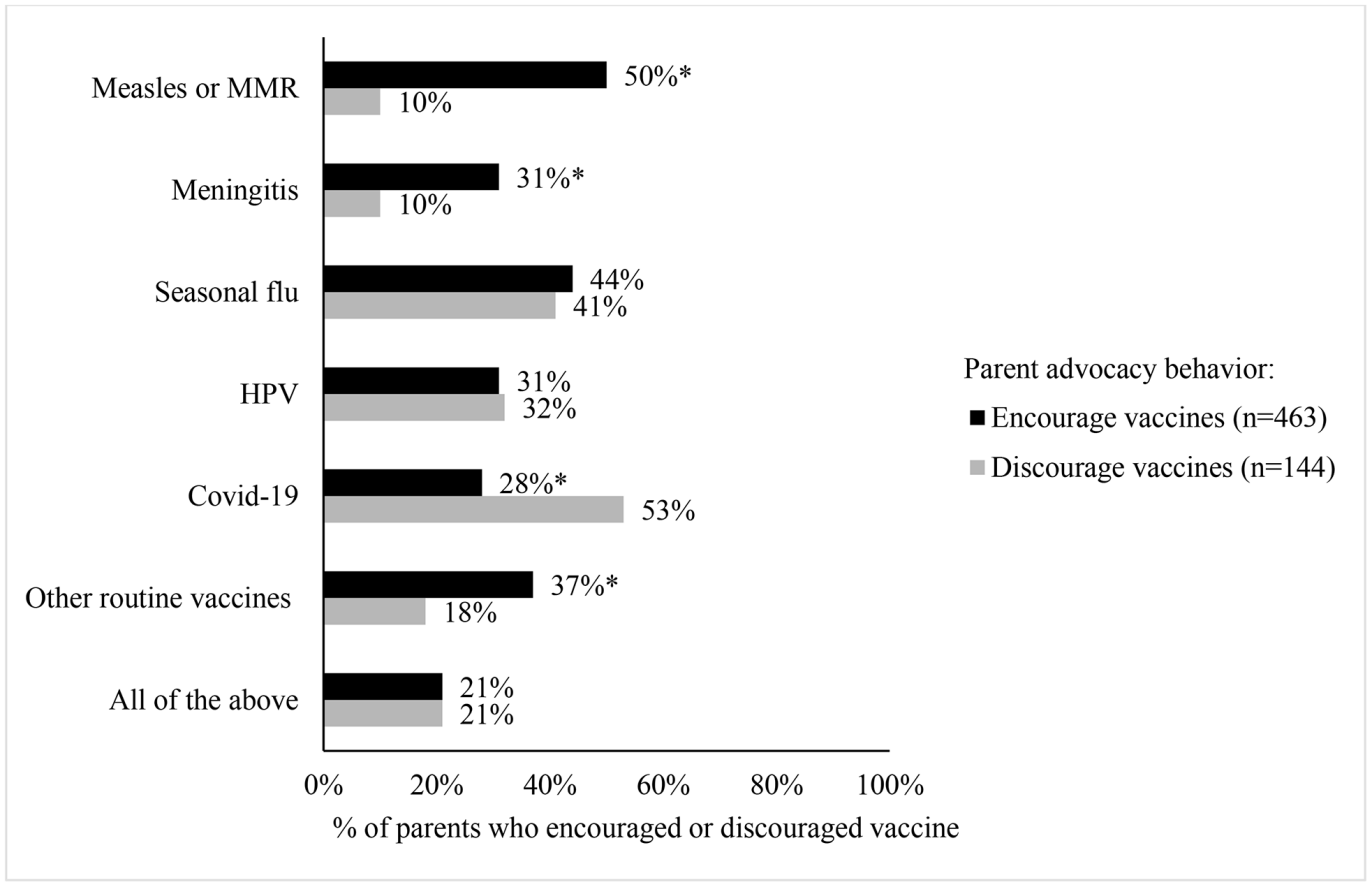
Routine vaccines parent advocates reported encouraging or discouraging to other parents. Reported as percentage of parents who encouraged or discouraged each vaccine type.

Higher proportions of vaccine encouragers (*n* = 463) than discouragers (*n* = 144) focused their advocacy on measles or MMR (50% vs. 10%) and meningitis vaccines (31% vs. 10%, both *p* < .05, Fig 1). In contrast, more discouragers than encouragers focused on Covid-19 vaccine (53% vs. 28%, *p* < .05). Encouragers and discouragers similarly focused on seasonal flu (44% and 41%), HPV (31% and 32%), and all routine vaccines for children (21% and 21%).

#### Audiences.

Both vaccine encouragers (*n* = 477) and discouragers (*n* = 149) most often reported that audiences for their advocacy included close friends (58% vs. 41%) and family members (57% vs. 42%), although encouragers reported engaging these audiences more often than discouragers (both *p* < .05, Table 3). Other audiences that encouragers and discouragers reported engaging were parents in their child’s school or community (27% and 22%), parents on social media (12% and 12%), and the broader public (7% and 13%).

**Table 3:** Audiences, strategies, and modes among parents who reported vaccine encouraging (*n* = 477) and discouraging (*n* = 151).

	Encouraging		Discouraging		
	*n*	(%)	*n*	(%)	*p*
Audiences^a^					
Close friends	282	(58)	66	(41)	*
Family members	273	(57)	67	(42)	*
Other parents in child’s school/community	135	(27)	35	(22)	
Other parents on social media	63	(12)	20	(12)	
Broader public	37	(7)	17	(13)	
Strategies^b^					
Share experiences getting vaccines for children	236	(47)	46	(27)	**
Share information about vaccine effectiveness	193	(38)	63	(42)	
Share advice or opinions about vaccines	181	(35)	57	(36)	
Share information about vaccine safety	167	(33)	59	(35)	
Share websites/other resources about vaccines	98	(20)	46	(31)	*
Share information about providers to go to	64	(13)	11	(5)	*
Modes^c^					
Conversations with other parents	334	(67)	69	(42)	**
Social media posts	82	(17)	32	(23)	
School or community advocacy	45	(8)	21	(15)	
State or national advocacy	19	(4)	13	(8)	*

*Note*. Table shows unweighted frequencies and weighted percentages. Items allowed multiple selections. Given the exploratory nature of the analysis, p-values were not corrected for multiple comparisons.

a Data missing for *n* = 2 parents who reported discouraging.

b Data missing for *n* = 5 parents who reported encouraging.

c Data missing for *n* = 1 parents who reported encouraging and *n* = 2 discouraging.

*p < 0.05.

**p < 0.001.

#### Strategies.

Higher proportions of vaccine encouragers (*n* = 472) than discouragers (*n* = 151) reported using the advocacy strategies of sharing their own experiences getting vaccines for children (47% vs. 27%) and information about which providers to go to (13% vs. 5%, both *p* < .05, Table 3). In contrast, more discouragers than encouragers shared websites or other resources (31% vs. 20%, *p* < .05). Encouragers and discouragers similarly reported sharing information about vaccine effectiveness (38% and 42%) and safety (33% and 35%), as well as their own advice or opinions about vaccines (35% and 36%).

#### Modes.

More vaccine encouragers (*n* = 476) than discouragers (*n* = 149) reported using conversations with other parents as an advocacy mode (67% vs. 42%, *p* < .05, Table 3). In contrast, more discouragers than encouragers used state or national advocacy (8% vs. 4%, *p* < .05). Encouragers and discouragers similarly used social media posts (17% and 23%) and school or community advocacy (8% and 15%).

### Advocacy identities

Parents who reported only encouraging vaccines (*n* = 399) most often endorsed the identities of vaccine advocate (43%), child health advocate (28%), and wellness advocate (24%, Table 4). These identities were also most common among parents who reported both encouraging and discouraging vaccines (*n* = 77) or neither behavior (*n* = 760): vaccine advocate (20% and 18%, respectively), child health advocate (18% and 18%), and wellness advocate (19% and 20%). In contrast, parents who reported only discouraging vaccines (*n* = 73) most often endorsed the identities of vaccine skeptic (45%), medical freedom activist (22%), and anti-vaccine activist (22%), in addition to child health advocate (23%).

**Table 4:** Vaccine advocacy identities endorsed by parents who only encourage other parents to vaccinate (*n* = 399), only discourage other parents (*n* = 73), both encourage or discourage (*n* = 77), or neither (*n* = 760).

	Only encourage		Only discourage		Both		Neither	
	*n*	(%)	*n*	(%)	*n*	(%)	*n*	(%)
Vaccine advocate	185	(43)	2	(3)	15	(20)	139	(18)
Child health advocate	120	(28)	20	(23)	14	(18)	152	(18)
Wellness advocate	106	(24)	20	(19)	16	(19)	160	(20)
Vaccine skeptic	10	(2)	35	(45)	6	(9)	123	(16)
Medical freedom activist	6	(1)	21	(22)	5	(5)	61	(8)
Anti-vaccine activist	3	(<1)	17	(22)	5	(7)	7	(1)
Vaccine influencer	19	(5)	3	(4)	7	(9)	6	(1)

Table shows unweighted frequencies and weighted percentages.

## Discussion

Over a third of US parents in our nationally representative study reported advocating for other parents to vaccinate their children, while only about one in ten discouraged other parents from vaccinating. Importantly, vaccine encouragers most often focused on measles vaccine while discouragers seldom did so, suggesting a potential asymmetry in the advocacy landscape favorable to public health during the recent measles resurgence. Overall, the two groups reported similar audiences, strategies, and modes, with encouragers especially likely to focus on family and friends, engage in conversations, and share vaccine experiences. Taken together, our findings suggest that vaccine encouragers deserve more attention than they typically receive, including as promising partners in efforts to increase coverage of MMR and other routine childhood vaccinations.

Our study highlights several understudied aspects of vaccine advocacy that can inform efforts to engage parents in vaccine promotion efforts. First, vaccine encouragers were far more common than discouragers, which has not to our knowledge been previously quantified. Indeed, extrapolating our findings to the US population suggests that there could be about ten million vaccine encouragers among parents of children ages 9–12 alone, which constitutes a large pool of potential partners [32]. Second, vaccine encouragers were relatively well represented across subgroups of parents, including by race/ethnicity and household income. Vaccination confidence emerged as the strongest correlate of encouraging vaccination, suggesting that parents’ vaccination beliefs may be more important than demographics for identifying supporters of routine childhood vaccination. Finally, vaccine encouragers often reported advocating at the interpersonal level by engaging friends and family. Prior research suggests that this type of peer-to-peer communication may be especially influential in informing parents’ vaccination behaviors [11,15,16]. In sum, our findings indicate that vaccine encouragers constitute a potential advocacy resource due to their large numbers, broad distribution, and integration into interpersonal social networks where their voices may be especially influential.

Our study also provides valuable data on parents who discourage vaccination and suggests a degree of nuance in advocacy behaviors. First and most notably, we were surprised to find that about half of vaccine discouragers also reported vaccine encouraging. This finding undercuts the common framing of advocates as entirely for or against vaccination and deserves additional research. Second, we observed relatively few discouragers. While this finding is reassuring, it is important to keep in mind that such parents can still be highly influential, especially to the extent they share negative vaccine information, which prior research shows can be more memorable and compelling than positive information [21]. Third, we found discouragers most often shared vaccine effectiveness and safety information and their own advice and opinions, but less often shared vaccine experiences compared to encouragers. Discouragers also more often engaged in state and national advocacy and less often had conversations with other parents, suggesting discouragers may be drawn to a larger anti-vaccine movement rather than engaging with their own social contacts. Thus, our study provides a new perspective for understanding how discouragers engage with other parents and the broader public, while also suggesting the need to move beyond conceptualizing advocacy as purely pro- versus anti-vaccination.

Relatedly, less than half of parents in our sample endorsed any of the prespecified identity terms we offered, suggesting parents may not readily adopt vaccine-related labels often used in the popular press and social media, even when they report advocacy behaviors. Prior research on group identities among pro- and anti-vaccination supporters suggests encouragers may have stronger overall identity endorsements compared to discouragers [33–35], but our results call into question how useful advocacy terms are for public health practitioners. Perhaps not surprisingly, vaccine advocate was the most commonly endorsed term among encouragers, and vaccine skeptic, medical freedom activist, and anti-vaccine activist were most common among discouragers. Yet, endorsement for more general terms including child health advocate and wellness advocate was consistent across advocacy behaviors, suggesting that those identities resonate with parents who both encourage and discourage vaccines. Given the lack of strong affinity for identity terms, efforts to categorize parents as pro- versus anti-vaccination when developing interventions should consider a degree of variation to avoid overly defining such groups. In terms of public health practice, it may be more effective to focus on the advocacy behavior of encouraging vaccines, rather than labeling parents with specific identity terms. In doing so, we can build on longstanding efforts to engage “fence sitters” and question our society’s trend towards polarizing vaccine beliefs [36].

Our findings suggest several avenues for engaging parents in interventions to increase coverage of MMR and other routine childhood vaccinations. Parents may need additional support to shift from interpersonal conversations with family and friends to broader advocacy as their mode, as encouragers were less likely to engage in state or national advocacy compared to discouragers. For example, efforts could leverage resources such as Vaccinate Your Family’s no-cost educational resource that teaches parents the basics of vaccine communication and safety and Voices for Vaccines’ “Vax Ambassadors” program which provides training sessions on advocacy-related topics and tools [37,38]. In light of our findings, public health leaders should consider ways to support these organizations in making vaccine advocacy opportunities more accessible for parents. We can also gain insight from previous research in the state of Washington that trained parent advocates who value immunization to engage in positive dialogue in their communities. The “Immunity Community” increased parents’ vaccine-related knowledge, vaccination confidence, and concern about other parents not vaccinating their children post-intervention [11]. While parents in our study similarly shared vaccine information and resources through conversations, those in the “Immunity Community” study also posted on social media and organized community-wide events to improve vaccine-related attitudes [11]. Given ongoing outbreaks of measles and other vaccine-preventable diseases, the time may be ripe to further promote social media use and organizing among parent advocates.

Our study also suggests several implications for future research. Vaccine encouragers may be conceptualized as an implementation strategy similar to clinical champions, or healthcare professionals who help their colleagues improve the delivery of evidence-based care in clinical settings [39]. As an opportunity for possible collaboration, clinics and parent-based organizations could consider pairing clinical champions with parents who might work together to improve interpersonal vaccine communication, with a specific focus on measles vaccination given the current outbreaks. Future research can also further support intervention studies through the development of validated measures to assess advocacy, building on a recently developed scale assessing advocacy behaviors for childhood vaccines among adults [40]. Lastly, future research should explore why parents might not report advocacy behavior including potential barriers such as lack of time and adequate resources or viewing vaccination as a provider’s responsibility to discuss, rather than parents’ themselves.

Our study has several strengths, including a large, nationally representative sample of parents that includes variation across key demographics, including parenting role, race/ethnicity, and political leaning. Limitations include the cross-sectional study design and use of self-reported data on vaccine advocacy behaviors. We acknowledge that we cannot determine causality in observed associations, such as those between vaccination confidence and advocacy behavior. Social desirability may have led some participants to overstate their involvement in vaccine encouraging or underreport discouraging, potentially inflating our encourager to discourager ratio. In addition, the terms “encouraging” or “discouraging” vaccines are not previously validated. While cognitive interviews of our survey items confirmed a common understanding, parents may have had differing interpretation of vaccine advocacy behavior depth or intensity. Given that our survey was fielded in May 2025, we characterized parental vaccine advocacy after the Covid-19 pandemic and during the start of a new government administration. Our findings should be considered in light of recent drops in public health trust, reduction of vaccine-related infrastructure, and highly publicized vaccine-preventable diseases which may have influenced the frequency of vaccine encouraging and discouraging compared to prior years [41–43]. Future studies may benefit from specifying a time frame for vaccine advocacy behaviors, as well as collecting data on additional variables, such as prior vaccination behavior and medical mistrust, to further assess potential correlates and confounders. Finally, our findings may be most generalizable to parents of children ages 9–12 years, as parents of younger or older children may have different advocacy priorities based on their child’s recommended vaccine schedule.

### Conclusions

This study furthers our understanding of the role of parent advocates in childhood vaccination and can help healthcare systems and public health organizations design future vaccine communication and behavioral interventions. Given that we found a considerable proportion of parents of children ages 9–12 years old reported encouraging routine vaccination for children, our study provides evidence for engaging parent vaccine advocates and may provide a model for other adolescent health initiatives. These results are notable during a time when the US risks losing its measles elimination status as more children are susceptible to vaccine-preventable diseases [4].

## Supporting information

S1 Table Bivariate correlates of encouraging and discouraging vaccination (*n =* 1,315). (DOCX)
