# Supplementary material for: Parents’ role as advocates for and against childhood vaccination: Findings from a nationally representative survey
Source: PLoS One. 2026 Jul 23;21(7):e0354147. doi: 10.1371/journal.pone.0354147 (PMC13395441; doi:10.1371/journal.pone.0354147)
Supplement: S1 Table — (DOCX) [file pone.0354147.s001.docx]

**S1 Table.** Bivariate correlates of encouraging and discouraging vaccination (*n*=1,315)

|  | No. of parents who encourage/ No. of parents in category (%) | | Bivariate | | No. of parents who discourage/ No. of parents in category (%) | | Bivariate | |
| --- | --- | --- | --- | --- | --- | --- | --- | --- |
|  |  |  | OR | (95% CI) |  |  | OR | (95% CI) |
| Parenting role |  |  |  |  |  |  |  |  |
| Mother | 310/804 | (39) | 1 |  | 89/804 | (11) | 1 |  |
| Father, grandparent or other | 167/511 | (32) | 0.71 | (0.53-0.93)* | 62/511 | (14) | 1.25 | (0.82-1.90) |
| Race/ethnicity |  |  |  |  |  |  |  |  |
| Non-Hispanic White | 294/803 | (35) | 1 |  | 76/803 | (10) | 1 |  |
| Non-Hispanic Black | 70/198 | (32) | 0.84 | (0.56-1.27) | 31/198 | (20) | 2.41 | (1.35-4.32)* |
| Hispanic | 72/201 | (37) | 1.06 | (0.72-1.55) | 30/201 | (16) | 1.75 | (1.03-3.00)* |
| Other | 41/113 | (41) | 1.29 | (0.81-2.04) | 14/113 | (11) | 1.22 | (0.60-2.47) |
| Educational attainment |  |  |  |  |  |  |  |  |
| Some college or less | 218/641 | (34) | 1 |  | 92/641 | (15) | 1 |  |
| Bachelor’s degree or higher | 259/674 | (39) | 1.26 | (0.97-1.64) | 59/674 | (8) | 0.49 | (0.33-0.74)* |
| Political leaning |  |  |  |  |  |  |  |  |
| Conservative | 109/446 | (24) | 1 |  | 65/446 | (14) | 1 |  |
| Moderate | 203/552 | (38) | 1.97 | (1.41-2.74)** | 68/552 | (15) | 1.01 | (0.65-1.58) |
| Liberal | 165/317 | (50) | 3.18 | (2.21-4.57)** | 18/317 | (5) | 0.34 | (0.18-0.64)* |
| Vaccination confidence |  |  |  |  |  |  |  |  |
| Low | 111/560 | (20) | 1 |  | 111/560 | (20) | 1 |  |
| High | 366/755 | (48) | 3.64 | (2.67-4.94)** | 40/755 | (6) | 0.27 | (0.17-0.43)** |
| Household income |  |  |  |  |  |  |  |  |
| Low | 113/295 | (37) | 1 |  | 49/295 | (18) | 1 |  |
| Medium | 108/344 | (32) | 0.80 | (0.52-1.22) | 42/344 | (13) | 0.68 | (0.39-1.20) |
| High | 256/676 | (37) | 1.02 | (0.71-1.46) | 60/676 | (10) | 0.52 | (0.32-0.85)* |

*Note*. Table shows unweighted frequencies and weighted percentages and odds ratios. OR: odds ratio. CI: confidence interval.

**p* < 0.05

***p* < 0.001
